# “It's Ok, We're Not Cousins by Blood”: The Cousin Marriage Controversy in Historical Perspective

**DOI:** 10.1371/journal.pbio.0060320

**Published:** 2008-12-23

**Authors:** Diane B Paul, Hamish G Spencer

**Affiliations:** Massachusetts Institute of Technology, United States of America

## Abstract

Marriage between first cousins is highly stigmatized in the West and, indeed, is illegal in 31 US states. But is the hostility to such marriage scientifically well-grounded?

In February, 2008, British environment minister Phil Woolas sparked a major row in the United Kingdom when he attributed the high rate of birth defects in the Pakistani community to the practice of marriages between first cousins. “If you have a child with your cousin, the likelihood is there'll be a genetic problem,” he told the *Sunday Times* [1]. Although a Muslim activist group demanded that Woolas be fired, he was instead promoted in October to the racially sensitive post of immigration minister. Most of his constituents would surely have shared Woolas' view that the risk to offspring from first-cousin marriage is unacceptably high—as would many Americans. Indeed, in the United States, similar assumptions about the high level of genetic risk associated with cousin marriage are reflected in the 31 state laws that either bar the practice outright or permit it only where the couple obtains genetic counseling, is beyond reproductive age, or if one partner is sterile. When and why did such laws become popular, and is the sentiment that informs them grounded in scientific fact?

US prohibitions on cousin marriage date to the Civil War and its immediate aftermath. The first ban was enacted by Kansas in 1858, with Nevada, North Dakota, South Dakota, Washington, New Hampshire, Ohio, and Wyoming following suit in the 1860s. Subsequently, the rate of increase in the number of laws was nearly constant until the mid-1920s; only Kentucky (1946), Maine (1985), and Texas (2005) have since banned cousins from marrying. (Several other efforts ultimately failed when bills were either vetoed by a governor or passed by only one house of a legislature; e.g., in 2000, the Maryland House of Delegates approved a ban by a vote of 82 to 46, but the bill died in the Senate.) The accompanying map (Figure 1) illustrates both the extent and the progress of legislation. It demonstrates that western states are disproportionately represented, reflecting the fact that either as territories or newly admitted states, they were writing their marriage codes from scratch and hence prompted to explicitly confront the issue. For the same reason, these states tended to be the first to prohibit cousin marriage.

**Figure 1:**
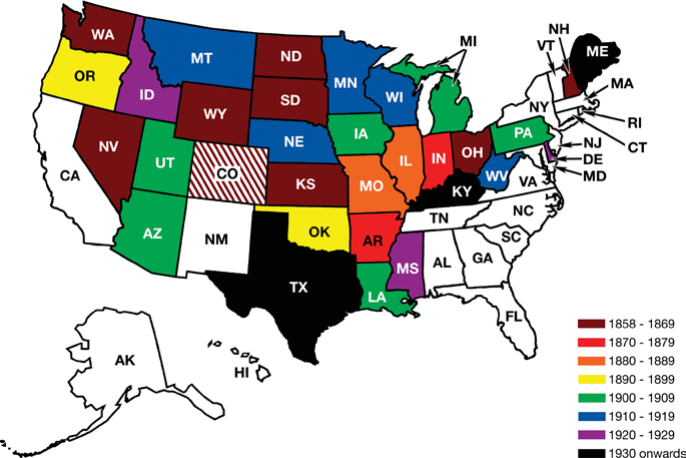
Map of the United States Showing States with Laws Forbidding First-Cousin Marriage Different colors reflect differences in the timing of passage of the laws. Colorado is shaded because its law was repealed. White states never had such bans.

Perhaps surprisingly, these bans are not attributable to the rise of eugenics. Popular assumptions about hereditary risk and an associated need to control reproduction were widespread before the emergence of an organized eugenics movement around the turn of the 20th century. Indeed, most prominent American eugenists were, at best, lukewarm about the laws, which they thought both indiscriminate in their effects and difficult to enforce [2]. In the view of many eugenists, sterilization of the unfit would be a far more effective means of improving the race.

Nonetheless, in both the US and Europe, the frequency of first-cousin marriage—a practice that had often been favored, especially by elites—sharply declined during the second half of the 19th century [3]. (The reasons are both complex and contested, but likely include improved transportation and communication, which increased the range of marriage partners; a decline in family size, which limited the number of marriageable cousins; and greater female mobility and autonomy [4,5].) The fact that no European country barred cousins from marrying, while many US states did and still do, has often been interpreted as proof of a special American animosity toward the practice [6]. But this explanation ignores a number of factors, including the ease with which a handful of highly motivated activists—or even one individual—can be effective in the decentralized American system, especially when feelings do not run high on the other side of an issue. The recent Texas experience, where a state representative quietly tacked an amendment barring first-cousin marriage onto a child protection bill, is a case in point.

The laws must also be viewed in the context of a new, post–Civil War acceptance of the need for state oversight of education, commerce, and health and safety, including marriage and the family. Beginning in the 1860s, many states passed anti-miscegenation laws, increased the statutory age of marriage, and adopted or expanded medical and mental-capacity restrictions in marriage law [7]. Thus, laws prohibiting cousin marriage were but one aspect of a more general trend to broaden state authority in areas previously considered private. And unlike the situation in Britain and much of Europe, cousin marriage in the US was associated not with the aristocracy and upper middle class but with much easier targets: immigrants and the rural poor. In any case, by the late nineteenth century, in Europe as well as the US, marrying one's cousin had come to be viewed as reckless, and today, despite its continued popularity in many societies and among European elites historically, the practice is highly stigmatized in the West (and parts of Asia—the People's Republic of China, Taiwan, and both North and South Korea also prohibit cousin marriage) [8–11]. The ironic humor of a New Zealand beer advertisement (Figure 2) nicely reflects current opinion in much of the world. But is the practice as risky as many people assume?

**Figure 2:**
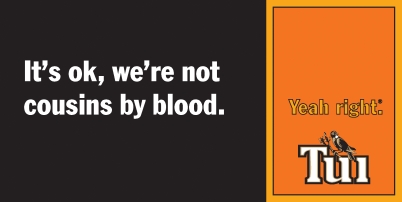
A Beer Advertisement from New Zealand, Part of a Humorous Series

Until recently, good data on which to base an answer were lacking. As a result, great variation existed in the medical advice and screening services offered to consanguineous couples [12]. In an effort at clarification, the National Society of Genetic Counselors (NSGC) convened a group of experts to review existing studies on risks to offspring and issue recommendations for clinical practice. Their report concluded that the risks of a first-cousin union were generally much smaller than assumed—about 1.7%–2% above the background risk for congenital defects and 4.4% for pre-reproductive mortality—and did not warrant any special preconception testing. In the authors' view, neither the stigma that attaches to such unions in North America nor the laws that bar them were scientifically well-grounded. When dealing with worried clients, the authors advised genetic counselors to “normalize” such unions by discussing their high frequency in some parts of the world and providing examples of prominent cousin couples, such as Charles Darwin and Emma Wedgwood [13].

In the aftermath of controversies ignited by Woolas's comments and similar remarks in 2005 by Labour Member of Parliament Ann Cryer, who asserted “We have to stop this tradition of first cousin marriages” [14,15], the NSGC report was cited by numerous scientific, legal, and lay commentators as testament to the low risk of cousin marriage and hence the lack of any compelling biological reason to avoid it. (Literally dozens of authors also asserted that the Darwins had ten healthy children—despite the deaths of three of them in infancy or childhood and Charles Darwin's own worries that consanguinity had affected the health and fertility of the intermarried Darwin and Wedgwood families, and of his and Emma's offspring in particular [2,16].) Although the report warned against generalizing from (and hence by implication to) more inbred populations, many writers, roughly averaging the statistics for birth defects and pre-reproductive mortality, noted that first-cousin marriage “only” increases the risk of adverse events by about 3%. But for several reasons, any overall calculation of risk is in fact quite complicated.

First, even assuming that the deleterious phenotype arises solely from homozygosity at a single locus, the increased risk depends on the frequency of the allele involved; it is not an immediate consequence of the degree of relatedness between cousins. (Interestingly, despite the British biometricians' harsh criticism of Mendelism, they were the first to describe this dependency in 1911 [17,18].) If a deleterious recessive allele has a frequency *q*, the ratio of the recessive phenotype in the offspring of first cousins relative to a panmictic population is (1 + 15*q*)/16*q*, which means the increase in risk is greater for *rarer* conditions [19]. For example, if *q* is 0.01, the ratio is 7.2; if *q* is 0.001, it is 63.4. Consequently, statistics on the risks associated with cousin marriage are necessarily averages across many traits, and they are likely to be different for different populations, which will often vary in the frequency of particular deleterious alleles. In the Pakistani immigrant population, for example, the quoted high average rate of birth defects may mask a single trait (or small number of traits) at very high frequency, a situation with different medical consequences from one characterized by a larger number of less-frequent disorders.

Second, children of cousin marriages are likely to manifest an increased frequency of birth defects showing polygenic inheritance and interacting with environmental variation. But as the NSGC report notes, calculating the increased frequency of such quantitative traits is not straightforward, and properly controlled studies are lacking. Moreover, socio-economic and other environmental influences will vary among populations, which can easily confound the effects of consanguinity. Inbred populations, including British Pakistanis, are often poor. The mother may be malnourished to begin with, and families may not seek or have access to good prenatal care, which may be unavailable in their native language [20]. Hence it is difficult to separate out genetic from socio-economic and other environmental factors.

Third, as the report also notes, the degree of increased risk depends on the mean coefficient of inbreeding for the population. That is, whether first-cousin marriage is an occasional or regular occurrence in the study population matters, and it is thus inappropriate to extrapolate findings from largely outbred populations with occasional first-cousin marriages to populations with high coefficients of inbreeding and vice-versa. Standard calculations, such as the commonly cited 3% additional risk, examine a pedigree in which the ancestors (usually grandparents) are assumed to be unrelated. In North America, marriages between consanguineal kin are strongly discouraged. But such an assumption is unwarranted in the case of UK Pakistanis, who have emigrated from a country where such marriage is traditional and for whom it is estimated that roughly 55%–59% of marriages continue to be between first cousins [21–23]. Thus, the usual risk estimates are misleading: data from the English West Midlands suggest that British Pakistanis account for only ~4.1% of births, but about 33% of the autosomal recessive metabolic errors recorded at birth [24]. However, for a variety of reasons (including fear that a cousin marriage would result in their being blamed for any birth defects), UK Pakistanis are less likely to use prenatal testing and to terminate pregnancies [20,25]. Thus the population attributable risk of genetic diseases at birth due to inbreeding may be skewed by prenatal elimination of affected fetuses in non-inbred populations. Moreover, the consequences of prolonged inbreeding are not always obvious. The uniting of deleterious recessives by inbreeding may also lead to these alleles being purged from the population. The frequency of such deleterious alleles, then, may be decreased, which (as shown above) means the relative risk is *greater*, even as the absolute risk decreases.

For all these reasons, the increased population-level genetic risks arising from cousin marriage can only be estimated empirically, and those estimates are likely to be specific to particular populations in specific environments. And of course for particular couples, the risks depend on their individual genetic makeup. It is also worth noting that both the increased absolute and relative risk may be relevant to assessing the consequences of consanguineous marriage. If the background risk of a particular genetic disorder were one in a million, a ten-fold increase in relative risk would likely be considered negligible, because the absolute increase is nevertheless minuscule. Conversely, the doubling of an absolute risk of 10% would surely be considered unacceptable. But the doubling of a background 3% risk may fall on a borderline, with the increase capable of being framed as either large or small. In any case, different commentators have certainly interpreted the same risk of cousin marriage as both insignificant and as alarmingly high.

Those who characterize it as slight usually describe the risk in absolute terms and compare it with other risks of the same or greater magnitude that are generally considered acceptable. Thus it is often noted that women over the age of 40 are not prevented from childbearing, nor is anyone suggesting they should be, despite an equivalent risk of birth defects. Indeed, the argument goes, we do not question the right of people with Huntington disease or other autosomal dominant disorders to have children, despite a 50% risk to offspring [26–29]. On the other hand, those who portray the risk as large tend to describe it in relative terms. For example, geneticist Philip Reilly commented: “A 7 to 8% chance is 50% greater than a 5% chance. That's a significant difference.” They also tend to compare the risk with others that are generally considered unacceptable. Thus a doctor asks (rhetorically): “Would anyone knowingly take a medication that has double the risk of causing permanent brain damage?” [30,31].

In closing, we note that laws barring cousin marriage use coercive means to achieve a public purpose and thus would seem to qualify as eugenics even by the most restrictive of definitions. That they were a form of eugenics would once have been taken for granted. Thus J.B.S. Haldane argued that discouraging or prohibiting cousin marriage would appreciably reduce the incidence of a number of serious recessive conditions, and he explicitly characterized measures to do so as acceptable forms of eugenics [32]. But Haldane wrote before eugenics itself became stigmatized. Today, the term is generally reserved for practices we intend to disparage. That laws against cousin marriage are generally approved when they are thought about at all helps explain why they are seemingly exempt from that derogatory label.

It is obviously illogical to condemn eugenics and at the same time favor laws that prevent cousins from marrying. But we do not aim to indict these laws on the grounds that they constitute eugenics. That would assume what needs to be proved – that all forms of eugenics are necessarily bad. In our view, cousin marriage laws should be judged on their merits. But from that standpoint as well, they seem ill-advised. These laws reflect once-prevailing prejudices about immigrants and the rural poor and oversimplified views of heredity, and they are inconsistent with our acceptance of reproductive behaviors that are much riskier to offspring. They should be repealed, not because their intent was eugenic, but because neither the scientific nor social assumptions that informed them are any longer defensible.
